# Comparison between two mobile pre-hospital care services for trauma patients

**DOI:** 10.1186/1749-7922-7-S1-S6

**Published:** 2012-08-22

**Authors:** Ricardo Alessandro Teixeira Gonsaga, Izabela Dias Brugugnolli, Gustavo Pereira Fraga

**Affiliations:** 1 Faculdades Integradas Padre Albino, Medical School, Department of Surgery, Catanduva, SP, Brazil; 2 Division of Trauma Surgery, Department of Surgery, School of Medical Sciences, University of Campinas, Campinas, SP, Brazil

## Abstract

**Objectives:**

Pre-hospital care (PH) in Brazil is currently in the phase of implementation and expansion, and there are few studies on the impacts of this public health service. The purpose of this study is to assess the quality of care and severity of trauma among the population served, using trauma scores, attendance response times, and mortality rates. This work compares two pre-hospital systems: the Mobile Emergency Care Service, or SAMU 192, and the Fire Brigade Group, or CB.

**Method:**

Descriptive study evaluating all patients transported by both systems in Catanduva, SP, admitted to a single hospital.

**Results:**

850 patients were included, most of whom were men (67.5%); the mean age was 38.5 ± 18.5 years. Regarding the use of PH systems, most patients were transported by SAMU (62.1%). The trauma mechanisms involved motorcycle accidents in 32.7% of cases, transferred predominantly by SAMU, followed by falls (25.8%). Regarding the response time, CB showed the lowest rates. In relation to patient outcome, only 15.5% required hospitalization. The average score on the Glasgow Coma Scale was 14.7 ± 1.3; average RTS was 7.7 ± 0.7; ISS 3.8 ± 5.9; and average TRISS 97.6 ± 9.3. The data analysis showed no statistical differences in mortality between the groups studied (SAMU - 1.5%; CB - 2.5%). The trauma scores showed a higher severity of trauma among the fatal victims.

**Conclusion:**

Trauma victims are predominantly young and male; the trauma mechanism that accounted for the majority of PH cases was motorcycle accidents; CB responded more quickly than SAMU; and there was no statistical difference between the services of SAMU and CB in terms of severity of the trauma and mortality rates.

## Introduction

Trauma injuries are becoming an increasing public health issue, especially in developing countries, whether due to their high mortality rates, or due to the high financial costs of treatment and recovery of these patients.

Reicheneim et al [1,2] classify violence in Brazil as the sixth highest cause of hospitalization, and the third highest cause of mortality. They found that young black men from poor communities are the principal victims, and also the principal offenders, in relation to community violence. In this country, the health authorities delegate responsibility for this service to the Fire Department, removing the health-related aspect of this attendance [3].

Several authors indicate that the pre-hospital mobile model practiced in nearly all western societies is inspired by the American and French systems [4,5]. The American system favors care carried out by paramedics (technicians), while the French favors the presence of doctors at the scene of the incident. Such systems usually have good results in terms of reducing morbidity and mortality, and neither model has been shown to be more effective than the other [3-7].

Brazil officially adopts the principles of the French model, the Mobile Emergency Care Service (MECS, or SAMU in Portuguese), adapting it to the local reality. The Brazilian Ministry of Health stipulates that critically ill or high-risk patients can only be removed from the scene of the accident in the presence of a full staff, including a doctor, travelling in an ambulance with advanced life support systems [8,9]. According to the Brazilian proposal, the population has two types of services at its disposal [9-11]: basic life support units (BLS, or UBS in Portuguese) with a paramedic (nursing technician) and advanced life support units (ALS, or USA in Portuguese), in which the minimum crew consists of a paramedic, a doctor and a nurse, together with intensive care equipment, the team members receiving guidance of doctors from central regulators [5,7].

In addition to SAMU, we also have the services of the Fire Department, through its “Rescue 193” (Fire Brigade Group - CB or “Resgate 193” in Portuguese). We are seeing a slow transition between the two services, one medicalized and with medical regulation, and the other driven by protocol.

In the city of Catanduva, which has a population of 112,820, there are two public pre-hospital healthcare services operating in the micro-region; one linked to the Municipal Health Department - the SAMU service - and the other to the Military Police Fire Department (CB) of the State Secretariat for Public Security Affairs of the State of São Paulo. These services work independently, acting in a loosely integrated way, but with no formal partnership between them at managerial level. Thus, there is a lack of practical action, when it comes to management, in the area of forming and improving the service, making best use of the training and experience of professional firefighters.

This study analyzes the APH performed by two different institutions; SAMU and CB, in the service to traumatized patients admitted to the only tertiary hospital belonging to the public health system in the municipality of Catanduva, in the state of São Paulo. This is probably the reality of pre-hospital care in various countries around the world, especially in terms of the resources used for this purpose. We therefore decided to study how the implementation of a new service affects the care of trauma patients.

## Material and methods

The Catanduva SAMU operates from a single base located in the center of the city, where three USB and one USA vehicles are housed. Two doctors are on call 24 hours a day, one of them being responsible for medical regulation and the other carrying out patient care when necessary. The teams were trained in 2006, following the guidelines established by the Ministry of Health [9]. The Catanduva CB operates from a single base located in the center of the city, where the USB vehicle is housed, together with vehicles for specialized use in various types of rescue and fire fighting; three firefighters are on call at all times.

The study involved two groups of individuals: the first consisted of patients treated in APH by the SAMU team, which were divided into two subgroups: SAMU - USB and SAMU - USA. The second group consisted of APH patients brought in by the CB team. The reference population was comprised of victims of traumatic injury aged 18 years or over.

All patients transported by SAMU or CB in the city of Catanduva during the period January 1^st^ to December 31^st^ 2007, and taken to a tertiary care hospital, were included in this analysis. Exclusion criteria were: patients transported to the hospital by other means, non-inclusion of the study parameters on the patient’s admission form; patients aged under 18; and patients who died on arrival in the emergency room (death on arrival).

The variables studied were: gender; age; type of injury; service that provided the pre-hospital care, and type of vehicle used to transport the patient; time T1, in minutes, from the initial call out of the arrival of the vehicle at the scene of the incident; time T2, in minutes, from the initial call out to the patient’s arrival at the hospital unit.

The following clinical data were evaluated and compared: the Revised Trauma Score (RTS) [12]; the Injury Severity Score (ISS) [13]; the probability of survival (Trauma and Injury Severity Score or TRISS) [14]; the causes of death and their classification. Deaths were classified as: preventable; potentially preventable (serious injuries, but not fatal, evaluation and treatment generally adequate, probability of survival less than 50% and greater than 25% or error in treatment, possibly influencing the outcome, directly or indirectly); and totally preventable [11].

The indices calculated were RTS, ISS and TRISS. The RTS was calculated based on the Glasgow Coma Scale (GCS), systolic blood pressure (SBP) and respiratory rate, the maximum value being 7.84. The ISS quantifies the severity of anatomical lesions in different body segments, with a maximum value of 75. Thus, ISS >10 represents moderate and severe anatomical lesions, while ISS >25 indicates very serious injuries. The TRISS represents the probability that the injured patient will survive, and is based on the RTS, ISS, patient’s age and type of injury (blunt trauma or penetrating trauma). The patients were grouped, according to their physiological condition, as normal (maximum RTS of 7.84) or altered (RTS with a loss of score in any of the three parameters). Patients with an ISS less than 10 were classified as having anatomical lesions of low severity, while those with an ISS greater than or equal to 10 were considered as having moderate or severe lesions (American College of Surgeons National Trauma Data Bank).

In relation to outcome, patients were classified according to survival (all patients who were discharged from the emergency unit - EU - or after hospitalization) or death (patients who died during the pre-hospital care and/or hospitalization).

A database was created using the program Epiinfo® version 3.5.1. The Kolmogorov-Smirnov statistical test was used to analyze the normality of the variables. For normal variables, the "student-t" and ANOVA tests were used, and for the non-parametric variables, the Fisher test (categorical variables) and Mann-Whitney test (common variables) were used.

The research project was approved by the Ethics and Research Committee under protocol no. CAAE 0015.0.218.000-09.

## Results

850 patients were selected for the study; the mean age was 38.5 ± 18.4 years and 67.5% (574 patients). The majority of the patients, 528 cases (62.1%) were attended by SAMU. Of these, 471 (89.2% used the USB and 57 (10.8% the USA. The CB, meanwhile, attended 322 incident call outs, comprising 37.9% of the total sample. In terms of the patients’ vital parameters, the mean Glasgow Coma Score was 14.8 ± 1.3, systolic blood pressure 129.9 ± 25 and respiratory rate 18.5 ± 3.9. The trauma severity scores were: RTS 7.7 ± 0.6, 3.8 ± 5.9 ISS, and the mean TRISS score was 98 ± 7.3. In relation to the mechanism of injury, the most frequent cause was accidents involving motorcycles, with 279 cases (32.8%), followed by falls, with 219 patients (25.8%). As a general trend within the sample, 123 patients (15.5%) required hospitalization, 702 (82.6%) were discharged from the emergency unit without hospitalization, and 16 (1.9%) died. 749 patients (88.1%) did not require surgery, and 101 (11.9%) did require surgery. The mean number of days that patients were kept under observation for more than 24 hours was 10.0 ± 9.3. The average time of pre-hospital care, in minutes, was 22.6 ± 10.

The group analyzed in this study consists of 850 patients who were transported by either SAMU or CB, in the city of Catanduva, during the one-year study period). The majority male (574 cases - 67.5%) with a mean age of 38.5 ± 18.5. It was observed that the age range was higher in patients attended by SAMU (35.8 ± 16.9 x 40.2 ± 19.2, *p* = *0.009*)*.* Analyzing the patient’s ages by type of transportation used (CB, USA and USB) it was observed that the average age of users who required USB (40.4 years) was higher when compared to users of other types of vehicles (CB = 35.8; USA = 37.9 years, respectively, p = 0.002).

Analyzing the type of pre-hospital care, most of the patients (528 cases - 62.1%) were attended by SAMU. Of the patients attended by SAMU, 471 (89.2%) used the USB and 57 (10.8%) the USA. CB attended 322 injured patients.

The most frequent type of injury involved motorcycles (32.7%), followed by falls (25.8%). Table 1 summarizes the data found. Analyzing the types of injury, SAMU attended more assaults (61 x 8, p<0.001) and falls (153 x 66, p = 0.005), while CB attended more motorcycle accidents (143 x 136, p < 0.001). Analyzing the different types of injury and the care provided to the patient by the SAMU vehicles, it was observed that in the vast majority of cases, the USB was used (57 x 471, p>0.001).

**Table 1:** Type of injury associated with pre-hospital mobile care systems and types of vehicles used.

Injury type	Total						
		CB	SAMU	*p 1*	USA	USB	*p 2*
Assault	69	8	61	*p* <*0.001*	5	56	*p* <*0.001*
Hit by vehicle	54	22	32	*p* = *0.652*	7	25	*p* = *0.113*
Automotive	88	36	52	*p* = *0.536*	12	40	*p* = *0.010*
Cycling accident	72	28	44	*p* = *0.848*	5	39	*p* = *0.975*
Stab wound	31	12	19	*p* = *0.913*	3	16	*p* = *0.773*
Motorcycle accident	279	143	136	*p* <*0.001*	12	124	*p* <*0.001*
Fall	219	66	153	*p* = *0.005*	11	142	*p* = *0.004*
Others	38	7	31	p = 0.009	2	29	*p* = *0.026*

Total	850	322	528		57	471	

In the analysis of times required for each call out by system, statistical differences were observed in all times, with CB showing short time intervals to deliver treatment (T1= 4.2 x 5.6, *p* <*0.001*, *T2* = *20.7 x 23.7*, *p*<*0.001*) compared to SAMU. In the analysis of time required for each vehicle, it was observed that the vehicles operated by CB had shorter times while the vehicles manned by USA teams had longer times (Table 2), with statistical differences in all the analyses (T1 = 4,2 x 5,6 min, p<0,001; T2 = 20,7 x 26,2 min, p<0,001).

**Table 2:** Treatment response times, by vehicle.

*Times calculated*	*Vehicle*	*Mean (min)*	*Variance*	*p*
T1	CB	4.22	10.23	
	SAMU – USA	5.60	34.24	
	SAMU – USB	5.59	13.70	*p* <*0.001*
T2	CB	20.69	118.56	
	SAMU – USA	26.16	235.28	
	SAMU – USB	23.45	66.70	*p* <*0.001*

It was observed that of the patients attended in this period, 702 (82.6%) were discharged from the EU after medical evaluation, 132 (15.5%) required hospitalization and 16 died (1.9%) (Table 3).

**Table 3:** Hospital conduct associated with the types of systems and vehicles used.

Conduct	Total	CB	SAMU				
			SAMU	*p 1*	USA	USB	*p 2*
Discharge from EU	702	258	444	*p* = *0.142*	26	418	*p* <*0.001*
Hospitalization	132	56	76	*p* = *0.244*	25	51	*p* <*0.001*
Death	16	8	8	*p* = *0.328*	6	2	*p* <*0.001*

Total	850	322	528		57	471	

		*χ^2^* = *2.53* *p* = *0.281*				*χ^2^* = *77.2* *p* <*0.001*	

Regarding the severity of trauma, the mean GCS score was 14.7 ± 1.3. ISS was 3.8 ± 5.9, RTS 7.7 ± 0.7 and TRISS 97.6 ± 9.3. Table 4 shows the data found for each study group and type of vehicle used. The data analysis shows no statistical differences between CB and SAMU. Analyzing the data separately by vehicle (p2), a difference is seen in all the trauma severity indices studied, with the USA attending patients with more severe traumas.

**Table 4:** Mean trauma score by system and vehicle used.

Severity	General						
		CB	SAMU	*p 1*	USA	USB	*p 2*
GCS	14.7	14.7	14.7	*p* = *0.381*	13.7	14.9	*p* <*0.001*
ISS	3.8	4.2	3.5	*p* = *0.132*	10.3	2.7	*p* <*0.001*
RTS	7.7	7.7	7.8	*p* = *0.503*	7.3	7.8	*p* <*0.001*
TRISS	97.6	97.9	98.0	*p* = *0.728*	91.6	98.9	*p* <*0.001*

Of the 132 patients who required hospitalization, the average hospitalization time, in days, was 8.9 ± 8.7, while in the analysis by system, no statistical differences were found (SAMU 8.8 days x CB 9.0 days, *p* = *0.916*). Neither were any statistical differences found in the analysis of pre-hospital care system (CB and SAMU) and patient outcome (CB - 314 x SAMU – 520, *p* = *0.164*).

Analyzing the 16 patients who died, there was no statistical difference between the mean ages (CB: 45.2 ± 22.9 years; SAMU: 54.9 ± 25.7; *p* = *0.441*), total PH time (CB: 35 ± 26.6 minutes; SAMU: 23 ± 6.0, *p* = *0.233*), RTS (CB: 5.6 ± 2.2; SAMU: 4.8 ± 3.3, *p* = *0.575*), ISS (CB: 28 ± 14.7; SAMU: 25.4 ± 14.2, *p* = *0.722*) and TRISS (CB: 70.6 ± 27.6; SAMU: 54.7 ± 44.0, *p* = *0.402*) in comparing the two types of PH (table 5). The mortality rate was 1.9% in the general sample, 1.5% for SAMU attendance and 2.5% for CB, with no statistical differences between the groups.

**Table 5:** Patient outcome according to the prognostic score.

Variable	Death	Survivors	*p*
RTS	5.2 ± 2.7	7.8 ± 0.2	*p* <*0.001*
ISS	26.7 ± 14.0	3.3 ± 4.7	*p* <*0.001*
TRISS	62.7 ± 36.5	98.7 ± 2.5	*p* <*0.001*
T1	6.4 ± 7.0	5.0 ± 3.7	*p* = *0.142*
T2	29 ± 19.6	22.5 ± 9.7	*p* <*0.05*

The comparison between the prognostic indices and APH times of patients who survived and those who died is shown in Table 5, in which the highest level of trauma severity is a fatal outcome. The only variable that showed no statistical difference was T1.

Table 6 shows the number of patients who died, detailing the type of trauma, the main injury, the cause of death, hospitalization time in days, prognostic indices, and inevitability of death. In the review of the medical records, the death of patient 13 was classified as preventable, because he had multiple fractures of the lower limbs without other significant injuries. During his hospitalization, the patient was confined to bed, and was not given any pharmaceutical prophylaxis for deep vein thrombosis in the first 48 hours postoperative (seventh day of hospitalization).

**Table 6:** Summary of deaths.

N	Age	System	T2	Type	Injury	Cause of Death	Days	RTS	ISS	TRISS	Death
**1**	73	CB	91	Automotive	FX leg	PE	30	7.84	9	99	Potential
**2**	19	USA	19	Bicycle	HT	HT	1	1.23	30	7	Inevitable
**3**	82	USB	18	Fall	FX femur	BCP	10	7.84	13	99	Potential
**4**	71	USA	29	Automotive	MC	BCP	23	7.55	34	78	Inevitable
**5**	22	CB	54	Burn	4^th^ degree	Cardiac	1	1.16	48	23	Inevitable
**6**	23	CB	40	Automotive	FX pelvis	BCP	18	5.14	34	69	Inevitable
**7**	23	USA	22	Motorcycle	Severe HT	HT	1	1.16	29	10	Inevitable
**8**	56	USA	16	Hit by vehicle	Severe HT	HT	1	1.16	50	2	Inevitable
**9**	78	CB	23	Fall	FX femur	PE	7	7.84	9	99	Potential
**10**	22	CB	23	Motorcycle	Vena cava	Shock	1	6.8	36	90	Inevitable
**11**	90	USB	21	Fall	FX femur	PE	4	7.84	9	99	Potential
**12**	44	CB	21	Automotive	Severe HT	BCP	45	5.96	34	85	Potential
**13**	51	USA	25	Automotive	FX multiple	PE	7	7.84	9	99	Preventable
**14**	60	CB	19	Fall	Severe HT	HT	8	5.6	25	54	Inevitable
**15**	47	USA	34	Automotive	Severe HT	BCP	60	3.51	29	44	Inevitable
**16**	40	CB	16	Motorcycle	Severe HT	HT	1	4.21	34	46	Inevitable

FX = Fracture; PE = Pulmonary Embolism; BCP = Bronchopneumonia; MC. = Myocardial Contusion; HT = Head Trauma

## Discussion

In relation to the patients’ age, it was observed that the data found are in agreement with national and international literature [14-16]. When we checked the behaviour of the age variable with respect to study groups, statistical differences were found, with SAMU showing a higher mean age than the individuals attended by CB. There is very little literature focusing on this type of analysis.

Carret et al [17], in a systematic review, sought to measure the prevalence of, and factors associated with the inappropriate use of emergency services. They found, among other factors, the difficulty of access to medical first aid, and concluded that first aid should be carried out in a qualified way. In fact, in the present study, the vast majority of patients in both study groups show trauma severity of low complexity, which may have been resolved by the first aid units (discharge from emergency units stands at over 81.7% of users). Deslandes et al [18] report that within a community, there is a local culture that seeks immediate attention and resolution to its ailments, associated with its own interpretation of what constitutes an emergency situation, leading to the use of all the available emergency care equipment and generating a burden of care in emergency care centers. In the city of Rio de Janeiro, O'Dwyer et al [19] analyzed the quality of care in emergency services and found misuse of these services in 65% of cases. It may be assumed that this situation also occurs in pre-hospital services, mainly due to the lack of medical regulation.

The literature is small and incipient when it comes to reporting the severity of users’ users. Marques et al [20] found, in the city of Porto Alegre (RS-Brazil), amongst patients treated by SAMU, an 8.2% utilization rate of the USA vehicle. In this study, the usage rate of the USA vehicle was 6.7% for the general study population study, and 10.8% for the SAMU users group. Nardoto [21], studying the victims attended by the air ambulance pre-hospital service, found a trauma severity score of 18.4%, based on the Glasgow Coma Scale, alone, showing that even for a vehicle that specializes in immediate care of critically ill patients, the rate of severity is relatively low.

Regarding the causes of injury, among those related to road traffic accidents, motorcycle accidents were the most prevalent (32.8%), followed by automobile accidents (10.3%). Gawryszewski et al [22], studying call outs to road traffic accidents in the State of São Paulo, observed that motorcycle accidents represented 29.8% of cases, followed by automobile accidents (25.7%) and then pedestrians being hit by vehicles (24.1%). In our study these figures were 32.8%, 10.3% and 6.3% respectively. The above authors conclude that motorcycle accidents account for most of the emergency calls and also that the victims were mostly young, male, low-skilled professionals, many of whom provide motorcycle taxi services that are widely used in urban areas of the state. The high prevalence of accidents involving road traffic has also been reported by several national and international authors [15,22-25].

Montenegro et al [27], studying mortality among motorcyclists in the Federal District (Brazil), found that over 70% of deaths occurred in hospitals. Furthermore they conclude that despite the severity of injuries, it is possible that the availability of emergency services and APH explain the lower proportion of deaths on public roads when compared to countries with disorganized public health systems. Marín-León et al [28], studying the trend of traffic accidents in Campinas (SP-Brazil), found an increase of 241% in the fleet of motorcycles in little more than a decade, representing almost 50% of all fatal accidents on public roads in 2008. In the present study motorcycles were involved in 32.8% of injury causes, rising to 56.7% when only road traffic accidents are considered, corroborating the above authors to conclude that multi-institutional actions are necessary to prioritize the prevention of motorcycle accidents.

A recently published study shows that violence and road traffic accidents account for almost two thirds of deaths of all trauma injuries [2]. In Brazil, homicide is listed as the most common cause of death, closely followed by road traffic accidents (36.4% and 29.3% respectively, in 2007). Mascarenhas et al [29] and Gawryszewski et al [30], analyzing emergency department visits due to traumatic injury in the Sentinel Services of Surveillance of Violence and Accidents system (VIVA), report that 10.4% of patient visits are motivated by violence, which affects more men than women. They also report a fact that draws attention, which is the means of transport used to get to the hospital: 25.2% of patients used private vehicles, and only 19.9% used a SAMU vehicle.

Also in relation to causes of injury, this study observed that 25.8% of patients were victims of falls, mostly being attended by SAMU. It is a fact that falls, and the resulting injuries, are more common among the elderly. Mello and Moyses [31], studying violence and accidents among the elderly, found in Curitiba (PR-Brazil) a prevalence of 22.5% of calls outs involving elderly patients, and that of these, 3.6% were victims of external causes.

Analyzing the pre-hospital transport systems, statistical differences were obtained for all the calculated times, with the CB showing shorter times in all the measurements (p<0.05). In fact, according to the working philosophy of this institution, these findings are within the expected range. The CB is heavily influenced by the North American system, which operates according to a working proposal of minimal intervention and maximum speed of transport. The proposal of SAMU, on the other hand, is to carry out medical procedures at the site of the accident, which increases the service time of the pre-hospital teams.

There was no statistical difference in mortality (*p* = *0.328*) between the SAMU (1.5%) and CB (2.5%) groups, this being an important index for analysis. There was no difference between the services of SAMU and of CB regarding hospitalization and deaths. Analyzing the data according to the type of vehicle used, there are statistical differences in deaths and hospital admissions associated with the use of the USA vehicle. In fact, in theory, more severe cases should be attended by this specialist team.

Other details that draw attention relate to levels of severity of the trauma. Amongst all the scores for trauma severity analyzed (GCS, ISS, RTS and TRISS), there were no statistical differences between the groups studied, either for the overall averages or for the grouping into classes. However, the same was not true in the analysis by type of vehicles; patients being treated by the USA vehicles showing the worst prognosis, according to the data found. A study conducted in Spain by Nieva et al [32] compared two models of emergency trauma care in two different towns: Pyrénées-Atlantiques (France) and Navarra (Spain). The authors found significant statistical differences in rescue times in APH, but comparable in-hospital mortality rates (*p* = *0.138*). In this study, the authors also report a statistical difference in the type of pre-hospital care; in France, according to the pre-hospital service index, 90.4% of patients receive direct care by an advanced support team, in medicalized ambulances or helicopters. In Spain, this index drops to 75.5% (*p*<*0.001*).

One of the pillars in trauma care is the presence of quality standards for the care provided. Coimbra et al [11] and Fraga [33] state that in Brazil, there is no organized system for trauma care that covers all the different phases of care. They report that there are no epidemiological studies, no records of trauma at municipal and state levels, a lack of information regarding pre-hospital care, and a lack of coordination between hospitals of different complexities and the Institute of Forensic Medicine, all of which pose barriers to a comprehensive study of the causes of death by external causes. In the present study, we analyze the patients who died. No statistical differences were found between the variables age, total time taken by the service, RTS, ISS and TRISS of patients attended by SAMU and CB. Unfortunately we do not have any data or information from other institutions that would enable a proper comparison with our data. This lack of statistical difference indicates that the pre-hospital system does not directly influence mortality, since there were no statistical differences, in this study, between the groups studied. When we look specifically at deaths, we see that the prognostic indices present statistical differences when compared with the survivors. Indeed, it is to be expected that the indices for patients who died will be different. Another parameter observed was time relative to APH. On analysis of the travel time of the vehicle to the location of the incident (T1) there was found to be no difference between the number of deaths and the number of survivors. This may be due to the fact that there is a perception that the urgency for the crew of the service vehicle is to arrive at the scene of the incident in order to identify the patient’s actual situation On analysis of the total service times, it was found that the patients who died showed the longest times, with a statistical difference between these and those who survived, due to the need for additional procedures, whether involving pre-hospital transport of the victim by CB, or the need for advanced procedures at the scene of the incident by the USA team.

The findings of this study were: the victims were mainly young, and male; motorcycle accidents accounted for the majority of cases; analysis of response times showed that CB had the shortest times; there were no statistical differences between SAMU and CB care in terms of trauma severity and outcome. Analysis by vehicle found statistical differences; the traumas suffered by patients who used the USA vehicle were more severe. As for mortality, there were no statistical differences between SAMU and CB. One preventable death was found, as well as five potentially preventable deaths and ten inevitable deaths. No relationship was found between patient complications and deaths and the type of service used in the pre-hospital care.

That said, it is observed that the implementation of SAMU occurred in Brazil, initially in a disordered fashion, and without integration with the various state devices, especially in the area of health. Currently there is a consensus that integration, especially of SAMU and CB, would optimize financial and human resources, as well as improving patient care and the outcomes for trauma patients. The process of assessing indicators and levels of injury should be continued, with professional training and control of service quality in all the phases of the service.

## Competing interests

The authors declare that they have no competing interests.

## Authors' contributions

GRAT was responsible for the study design, acquisition of data, statistical analysis, manuscript writing, and intellectual and scientific content of the study. BID was responsible for the acquisition of data. FGP was responsible for the applied methodology and critical revision of the manuscript.

## References

[B1] DATASUS [Internet page] 2011 Brazil. Presents health information and vital indicators from all the Brazilian cities, within various periods. Available at http://tabnet.datasus.gov.br. Accessed on February 1st, 2012.

[B2] Reicheheim ME Souza ER Moraes CL Jorge MHPM Furtado CM Silva P Violence and injuries in Brazil: the effect, progress made, and challenges ahead Lancet 2011 377 9781 1962 75 10.1016/S0140-6736(11)60053-6 21561649

[B3] Lopes SLB Fernandes RJ A brief review of medical prehospitalar care Medicina (Ribeirao Preto) 1999 32 381 7

[B4] Minayo MCS Deslandes SF Analysis of the implementation of a mobile pre-hospital treatment system in five Brazilian state capitals Cad Saúde Pública 2006 24 8 1877 86 10.1590/s0102-311x2008000800016 18709228

[B5] Scarpelini S Emergency and trauma system organization Medicina (Ribeirao Preto) 2007 40 3 315 20

[B6] Pinet LM Atención prehospitalaria de urgências em El Distrito Federal: las oportunidades del sistema de salud Salud Publica Mex 2005 47 1 64 71 10.1590/S0036-36342005000100010 15759915

[B7] Machado CV Salvador FGF O’Dwyer G Mobile Emergency Care Service: analysis of Brazilian policy Rev Saúde Pública 2011 45 3 519 28 10.1590/s0034-89102011005000022 21503554

[B8] Vieira CMS Mussi FC Implantation of the Emergency Ambulance Service in Salvador, Bahia: reality and challenges Rev Esc Enferm USP 2008 42 4 793 7 10.1590/S0080-62342008000400024 19192916

[B9] Brasil. Ministério da Saúde. Portaria n° 2.048/GM de 05 de novembro de 2002. Aprova o regulamento técnico dos sistemas de estaduais de urgência e emergência. Brasília - DF 2002 Available at http://portal.saude.gov.br/portal/saude/area.cfm?id_area=1787. Acessed February 1st, 2012.

[B10] Brasil. Conselho Federal de Medicina. Resolução CFM n° 1.671/03. Dispõe sobre o transporte inter-hospitalar de pacientes, diz sobre a classificação das ambulâncias de transporte, equipe profissional mínima para tal, responsabilidades e dá outras providências. Brasília - DF 2003 Available at http://www.portalmedico.org.br/resolucoes. Acessed February 1st, 2012.

[B11] Coimbra R Fraga GP Bansal V Constantini T Hoyt DB Controle de qualidade em trauma Ferrada R, Rodriguez A: Trauma - Sociedade Panamericana de Trauma 2010 Rio de Janeiro, Editora Atheneu 63 9

[B12] Champion HR Sacco WJ Copes WS Gann DS Gennarelli TA Flanagan ME A revision of the Trauma Score J Trauma 1989 29 5 623 9 10.1097/00005373-198905000-00017 2657085

[B13] Baker SP O'Neill B Haddon W Jr Long WB The injury severity score: a method for describing patients with multiple injuries and evaluating emergency care J Trauma 1974 14 3 187 96 10.1097/00005373-197403000-00001 4814394

[B14] Boyd CR Tolson MA Copes WS Evaluating trauma care: the TRISS method. Trauma Score and the Injury Severity Score J Trauma 1987 27 4 370 8 10.1097/00005373-198704000-00005 3106646

[B15] Batista SEA Baccani JG Silva RAP Guarda KPF Vianna RJA Jr Mechanisms of trauma, main injuries and severity of patients’ conditions in Catanduva - SP Rev Col Bras Cir 2006 33 1 6 10 10.1590/S0100-69912006000100003

[B16] Fraga GP Mantovani M Magna LA Trauma scoring in patients submitted to laparotomy Rev Col Bras Cir 2004 31 5 299 306

[B17] Carret MLV Fossa AG Domingues MR Inappropriate use of emergency services: a systematic review of prevalence and associated factors 2009 25 1 Cad Saúde Pública (Rio de Janeiro) 7 28 10.1590/s0102-311x2009000100002 19180283

[B18] Deslandes SF Minayo MCS Lima MLC Emergency care for victims of accidents and violence in Brazil Rev Panam Salud Publica 2008 24 6 430 40 19178782

[B19] O´Dwyer GO Oliveira SP de Seta MH Evaluation of emergency services of the hospitals from the QualiSUS program Cien Saude Colet 2009 14 5 1881 90 10.1590/S1413-81232009000500030 19851601

[B20] Marques GQ Lima MADS Ciconet RMI Conditions treated in the Mobile Medical Emergency Services in Porto Alegre - RS Acta Paul Enferm 2011 24 2 185 91 10.1590/S0103-21002011000200005

[B21] Nardoto EML Diniz JMT Cunha CEG The profile of victims attended by the Pernambuco prehospital air service Rev Esc Enferm USP 2011 45 1 237 42 10.1590/S0080-62342011000100033 21445514

[B22] Gawryszewski VP Coleho HMM Scarpelini S Zan R Jorge MHPM Rodrigues EMS Land transport injuries among emergency department visits in the state of São Paulo, in 2005 Rev Saúde Publica 2009 43 2 275 82 10.1590/S0034-89102009000200008 19287873

[B23] Peden M Scurfield R Sleet D Mohan D Hyder AA Jarawan E World report on road traffic injury prevention 2004 Geneva: World Health Organization

[B24] Bastos YGL Andrade SM Soares DA Characteristics of traffic accidents and victims treated through a pre-hospital service in a city in southern Brazil, 1997/2000 2005 21 3 Cad Saúde Pública, Rio de Janeiro 815 22 10.1590/s0102-311x2005000300015 15868039

[B25] Liberatti CLB Andrade SM Soares DA The new Brazilian traffic code and some characteristics of victims in southern Brazil Injury Prevention 2001 7 190 3 10.1136/ip.7.3.190 11565982 PMC1730749

[B26] Barros AJD Amaral RL Oliveira MSB Lima SC Gonçalves EV Motor vehicle accidents resulting in injuries: underreporting, characteristics, and case fatality rate 2003 19 4 Cad Saúde Publica, Rio de Janeiro 979 86 10.1590/s0102-311x2003000400021 12973564

[B27] Montenegro MMS Duarte EC Prado RR Nascimento AF Mortality of motorcyclists in traffic accidents in the Brazilian Federal District from 1996 to 2007 Rev Saúde Pública 2011 45 3 529 38 10.1590/s0034-89102011000300011 21552757

[B28] Marín-León L Belon AP Barros MBA Almeida SDM Restitutti MC Trends in traffic accidents in Campinas, São Paulo State, Brazil: the increasing involvement of motorcyclists Cad Saúde Pública 2012 28 1 39 51 10.1590/s0102-311x2012000100005 22267064

[B29] Mascarenhas MDM Silva MMA Malta DC Moura L Macário EM Gawryszewshi VP Epidemiological profile of violence patients of emergency help Services in the Injury Surveillance System Network in Sentinel Services (Viva) - Brazil, 2006 Epidemiol Serv Saúde, Brasília 2009 18 1 17 28

[B30] Gawryszewski VP Silva MMA Malta DC Kegler SR Mercy JA Mascarenhas MDM Violence-related injury in emergency departments in Brazil Rev Panam Salud Publica 2008 24 6 400 8 10.1590/S1020-49892008001200004 19178779

[B31] Mello ALSF Moysés SJ Situational analysis of the pre-hospital health services for attending accidents and violence against the elderly in Curitiba (PR, Brazil) Ciênc Saúde Coletiva 2010 15 6 2709 18 10.1590/s1413-81232010000600009 20922281

[B32] Nieva JLGS Boncompte MM Sucunza AE Louis CLJ Gómez MS Otano TB Comparison of mortality due to severe multiple trauma in two comprehensive models of emergency care: Atlantic Pyrenees (France) and Navarra (Spain) J Emerg Med 2009 37 2 189 200 10.1016/j.jemermed.2007.10.089 18829202

[B33] Fraga GP Quality programs on trauma care Medicina (Ribeirão Preto) 2007 40 3 321 8

